# Stochasticity in mammalian cell growth rates drives cell-to-cell variability independently of cell size and divisions

**DOI:** 10.1073/pnas.2516372123

**Published:** 2026-03-03

**Authors:** Ethan Levien, Joon Ho Kang, Kuheli Biswas, Scott R. Manalis, Ariel Amir, Teemu P. Miettinen

**Affiliations:** ^a^Department of Mathematics, Dartmouth College, Hanover, NH 03755; ^b^Department of Mechanical Engineering, Seoul National University, Seoul 08826, Republic of Korea; ^c^Institute of Advanced Machines and Design, Seoul National University, Seoul 08826, Republic of Korea; ^d^Koch Institute for Integrative Cancer Research, Massachusetts Institute of Technology, Cambridge, MA 02139; ^e^Department of Physics of Complex Systems, Weizmann Institute of Science, Rehovot 7610001, Israel; ^f^Department of Biological Engineering, Massachusetts Institute of Technology, Cambridge, MA 02139; ^g^Laboratory for Molecular Cell Biology, University College London, London WC1E 6BT, United Kingdom

**Keywords:** cell growth, cell size, cellular noise, cell divisions, heterogeneity

## Abstract

Cell growth rates exhibit cell-intrinsic cell-to-cell variability, which influences cell fitness and size homeostasis from bacteria to cancer. It remains unclear whether this variability arises from stochasticity in cell growth or division processes, or from cell-size-dependent growth regulation. To separate these potential sources of growth variability, single-cell growth rates need to be examined across different timescales. Here, we study cell size and growth regulation by tracking lymphocytic leukemia cell mass accumulation with high precision and minute-scale temporal resolution along long ancestral lineages. We first show that correlations between growth rates and cell-size nor asymmetric divisions explain cell-to-cell growth variability. We then isolate growth fluctuations by smoothing and detrending the growth rate dynamics using a Gaussian process regression. We find that these growth fluctuations drive cell-to-cell growth variability within ancestral lineages despite being independent of cell divisions, cell cycle, and cell size. Overall, our results provide a quantitative framework for understanding single-cell growth rates, and indicate that cell-intrinsic long-term patterns in growth are a byproduct of short-term growth fluctuations.

## Significance

Genetically identical cells can grow at different rates. This cell-to-cell growth variability can influence, for example, tumor biology, drug efficacy, and immune responses. Yet, the origin of this variability among genetically identical cells remains unresolved. Here, we track the growth of leukemia cells across different timescales to characterize patterns and fluctuations in cell growth. We find that cell-to-cell growth variability within cell lineages is explained by continuous, cell intrinsic noise in growth rates rather than size regulation or cell division specific growth changes. This suggests that, in the absence of cell-to-cell communication, cell-to-cell growth variability is primarily a byproduct of the stochasticity in metabolism.

All cell populations exhibit cell-to-cell variability in growth (1–8) and in the underlying growth driving processes, such as cell signaling, transcription, and translation (1, 4, 9–14). Understanding the variability between individual cells can elucidate many aspects of cell physiology and population dynamics. For example, information on cell-to-cell variability can help discriminate between different models of cell growth, gene expression, and size-control (9, 15–18). In bacteria and many cancers, cell-to-cell growth variability can also impact drug treatment responses, as nongenetic low-growth states, such as “persister” cells (19), are often more resilient to drugs (8, 17, 20, 21). Similarly, cell-to-cell variability in the rapid growth of immune cells can impact the resulting immune response (22). As surprisingly little is known about why cell growth varies between seemingly identical cells (23), understanding the magnitude of cell-to-cell growth variability and how this variability arises is of high importance.

A key requirement for studying cell-to-cell growth variability is precise growth measurements that enable growth rate monitoring on both short (within a cell cycle) and long (between generations) timescales. Knowledge about measurement precision is also required, as growth fluctuations could falsely arise from poor measurement precision or stability. Tracking single-cell volume growth rates over long timescales has previously been achieved in single-celled organisms using “mother machines” (1, 2). However, this is not the case for cell mass growth, and cell volume can fluctuate separately from mass (24). Furthermore, there are no long-term (≥4 generations) single-cell growth rate tracking data for mammalian cells, as existing mother machines are not suitable for mammalian cells. Here, we overcome these technical limitations by utilizing suspended microchannel resonators (SMR) to track the mass accumulation rate of leukemia cells across many generations. The SMR is a noninvasive buoyant mass sensor with ∼0.1% mass measurement precision (25–27). Under normal conditions, buoyant mass is analogous to dry mass (28), and mass accumulation can be considered as the sum of mass (i.e., nutrient) fluxes into and out of the cell (29). Because most of the cell’s dry mass is in macromolecules rather than ions and metabolites, buoyant mass acts as a proxy for cellular macromolecular content. From here on, we refer to buoyant mass simply as “mass.”

**Fig. 1.**
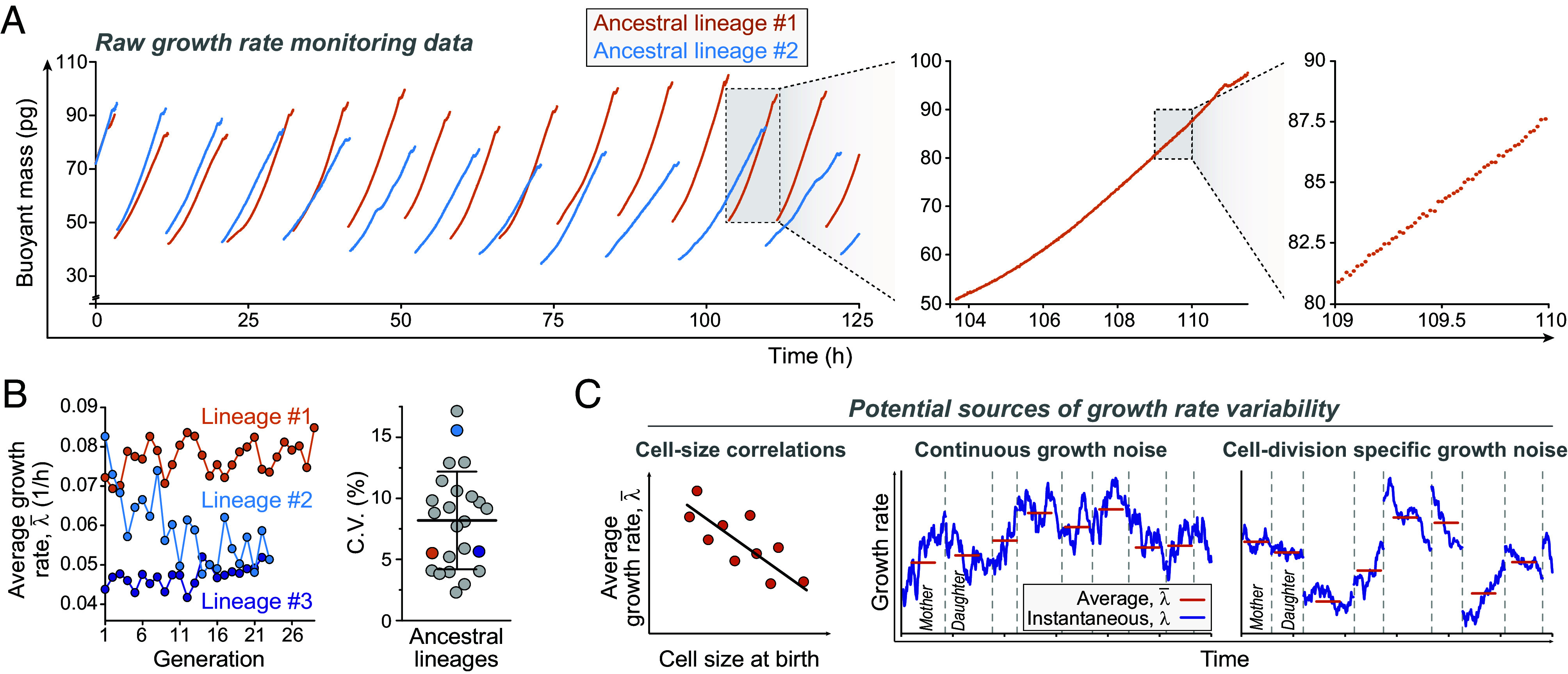
Cell-to-cell growth rate variability in ancestral lineages. (*A*) Two example sections of buoyant mass traces in ancestral L1210 cell lineages collected using the SMR. Buoyant mass of an isolated cell was measured approximately every minute under steady environmental conditions. At each division, one daughter cell is randomly discarded. Zoom-ins to a single cell cycle and to a 1-h section are shown on the *Right*. (*B*) *Left*, average cell growth rates ($\overset{¯}{\lambda }$) across the three longest lineages. *Right*, coefficient of variability (C.V.) of $\overset{¯}{\lambda }$ in all lineages (N=24 independent lineages). Bar and whiskers depict mean ± SD. (*C*) A schematic of the potential sources of cell-to-cell growth rate variability. Cell-to-cell growth rate variability may arise from cell-size-dependent growth. Alternatively, this variability could also arise from noise in cell growth. Such noise may be continuous or specific to cell divisions. Vertical dashed lines indicate cell divisions.

The SMR enabled us to monitor single-cell mass accumulation over timescales ranging from minutes to a week (Fig. 1*A*), allowing us to determine cell-to-cell growth variability (Fig. 1*B*). Using these cell mass (M) dynamics, we can obtain instantaneous growth rates (λ), and the averaged growth rates within each cell cycle ($\overset{¯}{\lambda }$). These are defined as (30)

[1] $$\begin{array}{c} \lambda =\frac{d}{\mathrm{dt}}\ln M\left(t\right),\;\text{(instantaneous growth rate)} \end{array}$$

and

[2] $$\begin{array}{c} {\overset{¯}{\lambda }}_{i}=\frac{1}{\tau_{i}}\ln\left(\frac{M(t_{i}+\tau_{i})}{M(t_{i})}\right),\;\text{(averaged growth rate)} \end{array}$$

respectively, where $t_{i}$ is the time at which the ith cell was born, $\tau_{i}$ is its cell cycle duration.

To understand cell-to-cell growth variability, we aim to identify deviations from strictly exponential growth in Eq. **1**. These deviations can arise from cell-size-dependent growth rates, which can reflect size-control (Fig. 1*C*) (31). Alternatively, deviations from exponential growth can also arise from noise in the biochemistry driving cell growth, resulting in continuous noise in growth rates (Fig. 1*C*) (4, 13, 32). If this noise is sufficiently large, it may influence cell-to-cell growth variability. However, noise in the underlying biochemistry of the cell can also result in unequal partitioning of cellular components during cell division (13, 32–35), which could impact cell-to-cell growth variability by imposing growth rate changes specifically at cell divisions (Fig. 1*C*). Therefore, a key question is whether continuous noise, i.e., fluctuations, in growth rates can be detected and whether they are “blind” to cell divisions.

Here, to disentangle these potential sources of cell-to-cell growth variability, we carry out a comprehensive characterization of the structure of mammalian cell growth (i.e., overlapping deterministic patterns, degree of randomness, timescale of growth fluctuations), which is currently largely undefined. Using high-precision, single-cell growth rate measurements of leukemia cells, we first show that most cell-to-cell growth variability is not explained by cell-size-dependent regulation of $\overset{¯}{\lambda }$ or division asymmetries. Next, we utilize Gaussian process (GP) regression analysis to infer and isolate growth fluctuations that are independent of longer timescale growth regulation as well as measurement fluctuations. These fluctuations reveal that continuous noise in growth, rather than cell division-specific changes, explains the majority of cell-to-cell growth variability in an ancestral cell lineage.

## Results

### Monitoring Cell Growth Across Ancestral Lineages.

Using the SMR, we collected a large dataset of mouse lymphocytic leukemia L1210 cell growth rates. We analyzed 24 lineages (each lineage is an independent experiment) with a total of 235 full cell cycles and 2,600 h of data (*SI Appendix*, Fig. S3*A* and Dataset S1). Optimization of system stability allowed us to monitor growth in ancestral lineages ranging from 3 to 29 full generations in length with cell mass measurements every ∼1 min. The SMR is not capable of tracking the growth of both sister cells following cell division, and one of the daughter cells is randomly discarded following every division. These data represent, to the best of our knowledge, the longest instantaneous growth rate monitoring data for mammalian cells to date. Furthermore, our measurements are not sensitive to volume fluctuations, which have been reported for many mammalian cells (36, 37).

We first characterized the overall growth behavior of the cells. The time that cells spent in the SMR did not correlate with $\overset{¯}{\lambda }$ (*SI Appendix*, Fig. S3*B*). The cell cycle durations (τ=10.2±2.0 h, mean ± SD) were comparable to independent studies with L1210 cells (25, 38), and the amount of variability in τ was comparable to a previous work where all data were collected at the same time (*SI Appendix*, Fig. S3*C*) (39). All L1210 cells in bulk culture were positive for the proliferation marker Ki-67 (*SI Appendix*, Fig. S3*D*) and all cells measured with SMR were growing. A cell in the SMR is in isolation from other cells and exposed to fresh media with every mass measurement, thereby excluding growth regulation due to autocrine and paracrine signals (40, 41). To focus our analyses on steady-state growth, the first (partial) cell cycle of each lineage was removed. We also examined the technical stability of our SMR setup by repeatedly measuring the mass of polystyrene beads. Bead measurements showed no systematic drift, and calibration remained stable within 0.07% over a typical cell cycle (*SI Appendix*, Fig. S3*E*). Overall, our ancestral lineage dataset reflects cancer cells growing under steady, high-growth conditions, where all cells actively proliferate, and growth behaviors can be attributed to cell-intrinsic sources.

When examining the generic characteristics of cell-to-cell growth variability, we first observed that lineage-to-lineage growth variability in the average growth rate was ∼11% (coefficient of variability, C.V.) (Fig. 1*B*, *Left* and *SI Appendix*, Fig. S3*A*). Experiments monitoring the proliferation rate of distinct L1210 cell lineages have also reported similar observations, where each cell lineage proliferates at its lineage-specific rate (8, 39). The cell-to-cell growth variability within each lineage was ∼8% (C.V.) (Fig. 1*B*, *Right*). Thus, the within-lineage variability explains approximately 1/3 of all cell-to-cell growth variance in our dataset. Here, we focus on explaining this within-lineage variability.

### Connections Between Size-Control and Cell-to-Cell Growth Rate Variability.

A potential reason for cell-to-cell growth variability is the need to maintain cell size homeostasis by adjusting growth rates in a size-dependent manner. Such cell-size-dependent growth has been reported in multiple cell lines (5, 42, 43). We therefore investigated how size homeostasis is achieved in our model system.

Cell size-control is often studied within an autoregressive framework (31, 44), where the cell size-control strategy is defined by a parameter α. This relates the cell’s birth mass ($M_{0}$) to the daughter cell’s birth mass ($M_{f}$):

[3] $$\begin{array}{c} M_{f}=2M_{0}\left(1-a\right)+2aE\left[M_{0}\right]+\xi_{M}, \end{array}$$

where $E[M_{0}]$ is the average initial mass, or equivalently the mass added during the cell cycle, and $\xi_{M}$ is a Gaussian random variable. Values of a range from 0 to 2 with lower a values indicating a weaker size homeostasis (44). Many cell types are reported to follow the adder model of size-control (a≈0.5) (5–7, 31, 45–49), where cells grow an approximately constant amount in each cell cycle.

The observed a can be achieved by regulating τ and/or $\overset{¯}{\lambda }$. Following ref. 5, we can reformulate the autoregressive model Eq. **3** on a log-scale, writing

[4] $$\begin{array}{c} M_{f}=M_{0}e^{\overset{¯}{\lambda }\tau }\Rightarrow \;\ln M_{f}=\;\ln M_{0}+\overset{¯}{\lambda }\tau \end{array}$$

Let $x=\;\ln M_{0}$. If the noise is small, the parameter a in Eq. **3** is given by $a\approx \alpha =-\mathrm{cov}(x,\overset{¯}{\lambda }\tau )/\mathrm{var}(x)$; see ref. 5. We use α over a because it is more natural statistics for identifying growth rate vs. generation time regulation.

Using our new dataset, we estimated α (Eq. **3**) in each lineage. On average, our data are consistent with an adder size-control strategy (α=0.55±0.06, mean ± SE, N=21, 3 shortest lineages excluded). Consistently, the mass added in each cell cycle was independent of cell birth mass in 8 of the 9 longest lineages in our dataset (*SI Appendix*, Fig. S4).

**Fig. 2.**
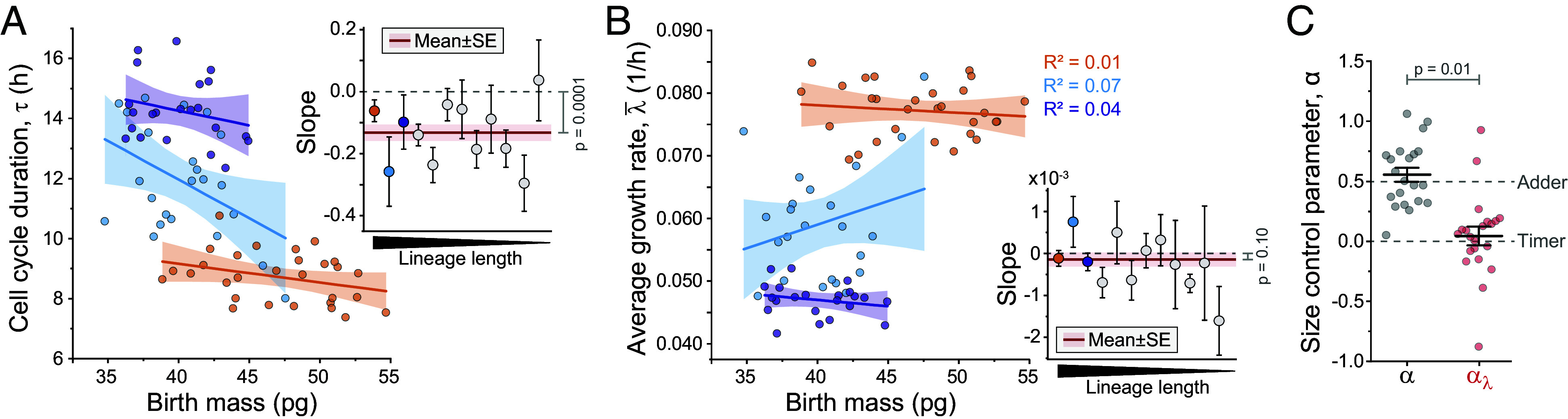
Size-control does not explain cell-to-cell growth variability. (*A* and *B*) Correlation between cell birth mass and τ (*A*) or $\overset{¯}{\lambda }$ (*B*) in the three longest lineages. Each *Inset* displays the slope of the correlation for all lineages with ≥8 cells (N=12 independent lineages). *Inset* red line and shaded area depict mean ± SE (data weighted by lineage length), *P*-value depicts one sample *t* test in comparison to 0. (*C*) Cell size-control parameter, α, when analyzed normally (α, gray) and when considering only the contribution of cell growth variability on α ($\alpha_{\lambda }$, red). Dots depict independent lineages (N=21 longest lineages); bar and whiskers depict mean ± SE, *P*-values obtained by Welch’s *t* test.

Nearly all lineages exhibited a negative correlation between cell birth mass and τ (Pearson $R^{2}=0.25\pm 0.07$, mean ± SE, Fig. 2*A*), but not between cell birth mass and $\overset{¯}{\lambda }$ (Pearson $R^{2}=0.10\pm 0.04$, mean ± SE, Fig. 2*B*). To quantify the relative contribution of $\overset{¯}{\lambda }$ to the overall size-control, we followed the approach of Cadart et al. (5) and used the scaled regression coefficient of initial size on average growth rate as a measure of the role of growth in size-control. The regression coefficient is given by

[5] $$\begin{array}{c} \beta_{x,\overset{¯}{\lambda }}=\mathrm{cov}\left(x,\overset{¯}{\lambda }\right)/\mathrm{var}\left(x\right). \end{array}$$

To obtain a nondimensional parameter, we scale by the average generation time to obtain $\alpha_{\lambda }=-\beta_{x,\overset{¯}{\lambda }}E\left[\tau \right]$. $\alpha_{\lambda }$ displayed values of approximately zero, indicating that growth rate regulation does not maintain size-control (Fig. 2*C*). Thus, in L1210 cells, cell size homeostasis could be explained by the regulation of τ alone, and the variability in cell-to-cell growth rates cannot be explained by size-control.

### Cell-to-Cell Growth Variability Is Decoupled from Cell Division Symmetry.

**Fig. 3.**
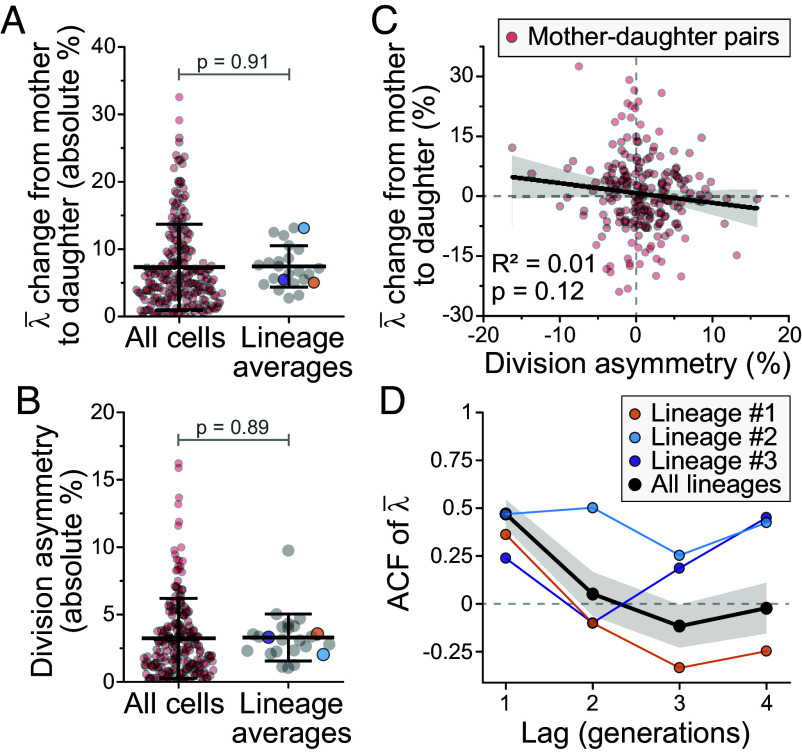
Cell-to-cell growth variability is independent of cell mass division errors. (*A* and *B*) Absolute % change in $\overset{¯}{\lambda }$ from mother to daughter (*A*), and absolute % cell mass division asymmetry (i.e. deviation from perfectly symmetrical division of cell mass) (*B*). Data are shown separately for all cells (red, n=211 mother-daughter pairs) and the averages of each lineage (gray, N=24 independent lineages, colored dots depict the three longest lineages). Bar and whiskers represent mean ± SD, *P*-values obtained by Welch’s *t* test. (*C*) Correlation between cell mass division asymmetry and the change in $\overset{¯}{\lambda }$ from mother to daughter. Black line and shaded area depict linear fit and 95% CIs. Pearson correlation and *P*-value (ANOVA) are also displayed (n=221). (*D*) Autocorrelation of $\overset{¯}{\lambda }$ in the three longest lineages (colored) and the average of the 10 longest lineages. Black line and shaded areas represent mean ± SEM. With a lag of 1 generation, the autocorrelation was significantly different from zero (P=0.0001, One Sample *t* test comparison to 0). With a lag ≥2 generations, the autocorrelation was indistinguishable from zero (P≥0.3).

We next examined if asymmetric cell mass divisions, where the mass of the newborn daughter cell is not perfectly 1/2 of the mass of the dividing mother cell, could explain the cell-to-cell growth variability. In each lineage, $\overset{¯}{\lambda }$ typically changed ∼7% from mothers to daughters (Fig. 3*A*), and cell mass divisions displayed, on average, ∼3% deviation from perfectly symmetrical divisions (Fig. 3*B*). When we performed a regression on the division asymmetry with the change in $\overset{¯}{\lambda }$ in each mother-daughter pair as the response variable, we did not observe any correlation (Fig. 3*C*). We note that this degree of division asymmetry is very low, and previous results have suggested over twofold higher asymmetry in cell mass divisions across cell lines (50). In addition, the lineages displayed little memory of $\overset{¯}{\lambda }$ and the autocorrelation function of $\overset{¯}{\lambda }$ decayed to zero in two generations (Fig. 3*D*). However, this conclusion is only reflective of lineages on average, as we observed significant lineage-to-lineage variability in the memory of $\overset{¯}{\lambda }$. Overall, we conclude that the variability in $\overset{¯}{\lambda }$ is not explained by systematic patterns (memory) in $\overset{¯}{\lambda }$ or by asymmetric cell mass divisions.

### Isolation of Growth Fluctuations.

Having excluded cell size and mass division asymmetry as potential explanatory variables for the growth rate fluctuations, we moved to examine the structure of growth within the cell cycle in more detail. As discovered in a previous study (51), we observed that each lineage had a cell-cycle-dependent growth trend that was dependent on the relative age in the cell cycle, not the absolute time since cell birth (*SI Appendix*, Fig. S5). This results in λ being cell-size and cell-cycle-dependent. To isolate fluctuations in λ around the cell-cycle-dependent growth trend, we used a Gaussian process (GP) regression analysis. GP is a Bayesian, kernel-based method which can be used to simultaneously smooth and decompose a function f, as shown before using different types of growth measurements (52, 53). The GP is based on a series expansion of the form $f\left(t\right)=\sum_{i\geq 0}\beta_{i}\phi_{i}\left(t\right),$ where $\left\{\phi_{i}\left(t\right)\right\}$ represent basis functions. Instead of specifying these functions directly, one places Gaussian priors on $\beta_{i}$. The posterior distribution $p(f\left(t\right)|\left\{\hat{f}\left(t_{i}\right)\right\})$ (the distribution of the function values f conditioned on the data) can then be obtained analytically by marginalizing over the $\beta_{i}$. This distribution is determined only by the covariance function

[6] $$\begin{array}{c} k\left(t,t^{\prime }\right)=\mathrm{cov}\left(f\left(t^{\prime }\right),f\left(t\right)\right) \end{array}$$

which is selected based on assumptions about the signal. The sum and derivatives of a Gaussian process are also Gaussian processes, and the process inherits the smoothness properties of the kernel (54). Consequently, we can use the isolated mass terms of GP to infer λ. Importantly, in GP, the measurement errors do not propagate, allowing us to avoid spurious correlations in the inferred λ.

**Fig. 4.**
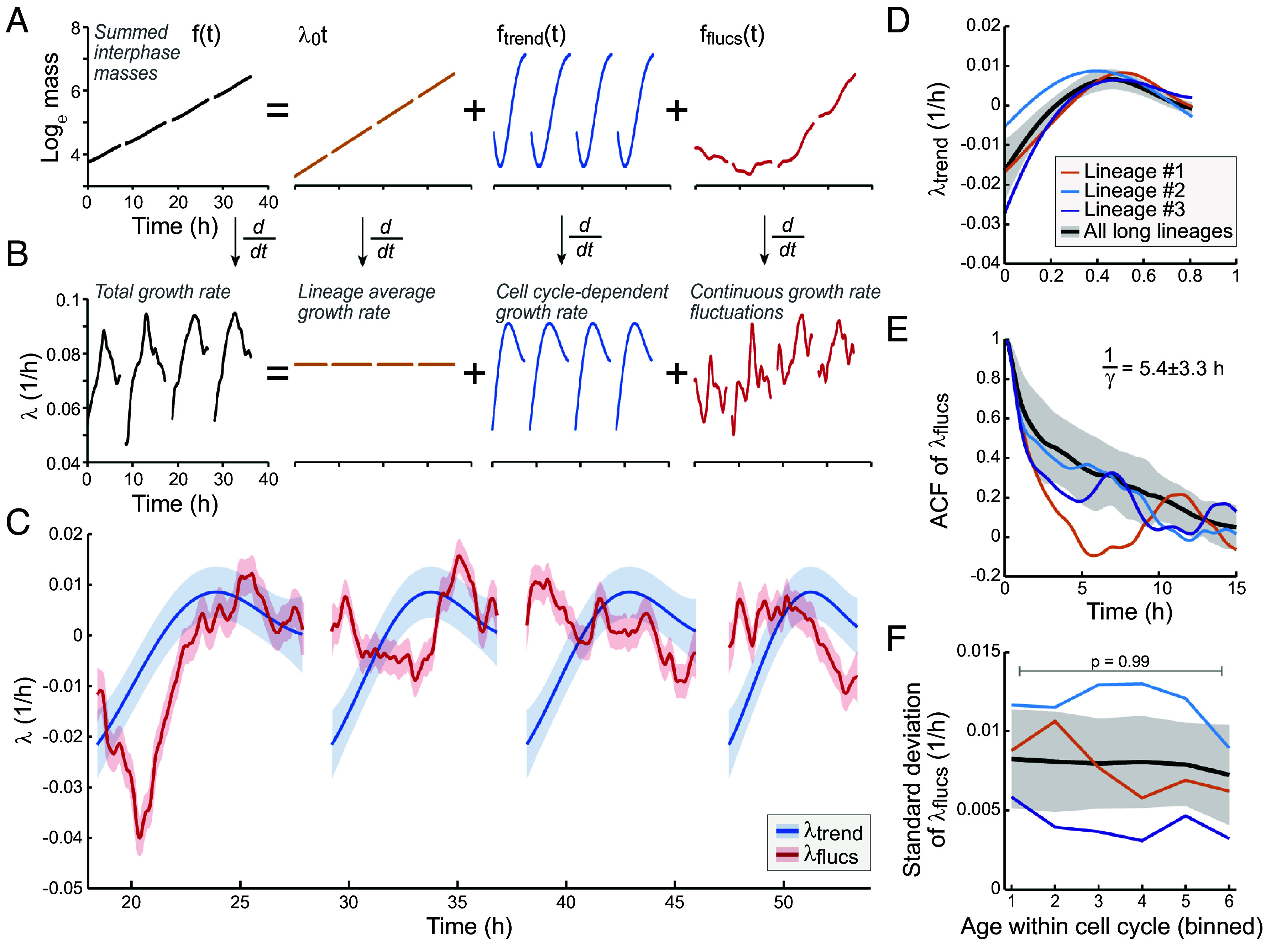
Growth rate fluctuations are independent of the cell cycle. (*A* and *B*) Analysis workflow: (*A*) Cell mass traces are plotted on a logarithmic scale; mitoses are removed and the mass traces are summed. Gaussian process is used to decompose the summed masses into discrete terms that reflect the lineage average mass trend (orange), cell-cycle-dependent mass trend (blue), and continuous mass fluctuations (red). (*B*) The isolated mass terms are converted to instantaneous growth terms. Note that the decomposed terms are plotted on different y-axis for illustration purposes. (*C*) An example of the isolated instantaneous growth terms over four full cell cycles in lineage #1. The gaps in data are cell divisions. Solid lines and shaded areas display the mean ± SD of each posterior distribution. The data are normalized to mean-zero. (*D*) The cell-cycle-dependent λ. (*E*) Autocorrelations of the λ fluctuations. The relaxation time is shown as mean ± SD. (*F*) Magnitude (SD) of the λ fluctuations at different cell ages. *P*-value obtained by ANOVA. In panels (*D*–*F*), colored lines depict the three longest lineages and black line with shaded areas depict all lineages (mean ± SD, N=10 longest lineages).

Our specific GP pipeline (*SI Appendix*, section 2) is as follows (Fig. 4*A*): First, we paste together the log masses to obtain a continuous signal:

[7] $$\begin{array}{c} \hat{f}\left(t\right)=\;\ln\frac{M_{i}\left(t\right)}{M_{i}^{b}}+\sum_{j<i}\ln\frac{M_{j}^{d}}{M_{j}^{b}}, \end{array}$$

where $M_{i}^{b}$, $M_{i}^{d}$, and $M_{i}\left(t\right)$ are the masses of the ith cell in a lineage at cell birth, cell division, and (absolute) time t, respectively. Due to the complex growth dynamics in mitosis (27, 28), we excluded mitoses from our analysis. Our GP decomposes the log summed mass measurements as

[8] $$\begin{array}{c} f\left(t\right)=\lambda_{0}t+f_{\mathrm{trend}}\left(t\right)+f_{\mathrm{flucs}}\left(t\right)+\epsilon \left(t\right), \end{array}$$

where ϵ is a delta correlated Gaussian noise term.

This GP-based approach enabled us to isolate cell mass and λ behaviors (Fig. 4 *A* and *B*). The posterior distributions of the cell-cycle-dependent $\lambda_{\mathrm{trend}}=f_{\mathrm{trend}}^{\prime }\left(t\right)$ and the fluctuations $\lambda_{\mathrm{flucs}}=f_{\mathrm{flucs}}^{\prime }\left(t\right)$ allowed us to separate changes in these growth behaviors on a subhour timescale (Fig. 4*C*). Before analyzing these growth behaviors in more detail, we verified that the cell mass and λ fluctuations reflect changes in cell mass rather than measurement error. We found that consecutive residuals of our GP analysis were not correlated (*SI Appendix*, Fig. S6*A*, $R^{2}=0.0046$). The degree of these correlations was comparable to the degree of correlations observed in polystyrene bead measurements (*SI Appendix*, Fig. S6*B*), which is the level expected based purely on measurement noise. However, we note that lineage # 2 displayed higher correlations between consecutive residuals than other lineages.

We also observed that the GP-isolated mass fluctuations in cells are orders of magnitude higher than those observed in polystyrene beads (*SI Appendix*, Fig. S6 *C* and *D*) and that the cell mass fluctuations took place on several orders of magnitude longer timescales than the bead mass fluctuations (*SI Appendix*, Fig. S6*E*). Thus, the GP-isolated cell mass and λ fluctuations do not reflect measurement artifacts but rather “true” growth behaviors.

### Growth Fluctuations Are Independent of the Cell Cycle and Cell Size.

We examined the GP-isolated growth terms to understand their impact on λ. The cell-cycle-dependent $\lambda_{\mathrm{trend}}$ was typically maximized in the middle of each cell cycle (at age 0.5 ± 0.1, mean ± SD, Fig. 4*D*). From cell birth to the point of maximum growth, the cell cycle influenced λ by 0.028 ± 0.008 1/h (mean ± SD), which corresponds to 38 ± 14% (mean ± SD) of total λ. A similar conclusion was reached when growth rates were analyzed using a simple fitting approach (*SI Appendix*, Fig. S7). The influence that the $\lambda_{\mathrm{trend}}$ and the $\lambda_{\mathrm{flucs}}$ had on the total λ were comparable. However, as our GP analysis assumes every cell in a given lineage displays the same $\lambda_{\mathrm{trend}}$ (after normalization for cell age), essentially all cell-to-cell growth variability is included in the $\lambda_{\mathrm{flucs}}$. This allows us to focus on the $\lambda_{\mathrm{flucs}}$ as we aim to understand cell-to-cell growth variability.

When examining the autocorrelation of $\lambda_{\mathrm{flucs}}$, we did not observe any periodicity (Fig. 4*E*). This is in contrast to a recent study, which also examined mass growth in L1210 cells, proposing that cell growth undergoes oscillations (55). The $\lambda_{\mathrm{flucs}}$ displayed a relaxation timescale (1/γ) of ∼5 h whether analyzed from the autocorrelation function or from the variance in the magnitude of the fluctuations (*SI Appendix*, section 1). These relaxation timescales are shorter than τ and thereby consistent with $\overset{¯}{\lambda }$ having little memory over generations.

Next, we looked for cell cycle dependency in $\lambda_{\mathrm{flucs}}$. We did not observe any difference in the magnitude (SD) of $\lambda_{\mathrm{flucs}}$ between the cells of different age in the cell cycle. Thus, the $\lambda_{\mathrm{flucs}}$ are independent of the cell cycle state (and approximate cell size) (Fig. 4*F*), even though the total growth rate of cells is not (Fig. 4*B*). Previous studies in bacteria and mammalian cells have suggested that cell volume growth variability is higher in small cells at the beginning of the cell cycle than in large cells later in the cell cycle (30, 36). As we do not observe this, cell cycle-dependent cell mass behavior might be distinct from cell cycle-dependent volume behavior. To further analyze if the $\lambda_{\mathrm{flucs}}$ displayed cell-size and/or growth dependency, we performed regressions on the magnitude of the $\lambda_{\mathrm{flucs}}$ with cell’s birth mass, division mass, the mass added in a cell cycle or τ (*SI Appendix*, Fig. S8). We did not observe any statistically significant correlations. Overall, these results suggest that the $\lambda_{\mathrm{flucs}}$ do not reflect cell-size or cell-cycle-dependent growth regulation, nor do the $\lambda_{\mathrm{flucs}}$ display any clear patterns.

We have also used our data to analyze cell-to-cell variability in λ on different timescales. We detail this analysis and its potential impact in *SI Appendix*, section 3.

### Cell-to-Cell Growth Variability Is Predicted by Cell Division-Independent Growth Fluctuations.

Our isolation of $\lambda_{\mathrm{flucs}}$ provides us with an opportunity to examine if cell growth rates are sensitive to cell division events. We compared the observed cell-to-cell variability in $\overset{¯}{\lambda }$ to the prediction of a simple null model in which instantaneous growth rate fluctuations are causally decoupled from the cell cycle. Specifically, we modeled $\lambda_{\mathrm{flucs}}\left(t\right)$ as an Ornstein–Uhlenbeck (OU) process—essentially a continuous time mean-reverting random walk (56)—which was motivated by the exponential decay in $\lambda_{\mathrm{flucs}}$ autocorrelations and the lack of correlations between $\lambda_{\mathrm{flucs}}$ and cell size or age (Fig. 4*E* and *SI Appendix*, Fig. S8). An OU process can be defined mathematically by the stochastic differential equation

[9] $$\begin{array}{c} \frac{d}{\mathrm{dt}}\lambda_{\mathrm{flucs}}=-\gamma \lambda_{\mathrm{flucs}}+\sqrt{2D}\xi . \end{array}$$

Here, ξ is a white noise term, D is the diffusion coefficient and γ is the relaxation rate. At cell division, we assumed that $\lambda_{\mathrm{flucs}}$ is perturbed by a Normal random variable z. While in reality noise at divisions may be coupled to cell size, the correlations with size only manifest in higher-order terms, which are excluded from the minimal model, and are likely not detectable with the quantity of data we have.

This model interpolates between two simple limits: As the growth noise introduced at cell division ($\sigma_{z}$) →0 we are left with the standard OU process which is “blind” to cell divisions (continuous noise). As D→0, we obtain a model where growth variation is tied to the cell division events. The contribution of the continuous growth fluctuations and division-specific growth noise can be identified by looking at the time averaged growth rate,

[10] $$\begin{array}{c} {\overset{¯}{\lambda }}_{t}=t^{-1}\ln M\left(t\right)/M\left(0\right), \end{array}$$

where t=0 is when cell division occurs. The variance of ${\overset{¯}{\lambda }}_{t}$ can be decomposed as

[11] $$\begin{array}{c} \mathrm{var}\left({\overset{¯}{\lambda }}_{t}\right)=\sigma_{c,t}^{2}+\sigma_{d,t}^{2}, \end{array}$$

where $\sigma_{c,t}^{2}$ and $\sigma_{d,t}^{2}$ respectively represent additive contributions to the overall variation from the OU and division noise.

When the characteristic relaxation timescale (1/γ) of this process is much less than the cell age (the absolute time since cell birth), these are given by

[12] $$\begin{array}{c} \sigma_{c,t}^{2}\approx \frac{2D}{\gamma^{2}t}. \end{array}$$

and

[13] $$\begin{array}{c} \sigma_{d,t}^{2}\approx \frac{\sigma_{z}^{2}}{{\left(\gamma t\right)}^{2}}. \end{array}$$

suggesting that the scaling of the variance in $\overset{¯}{\lambda }$ with time can be used to distinguish between continuous growth noise and division noise (*SI Appendix*, section 1).

**Fig. 5.**
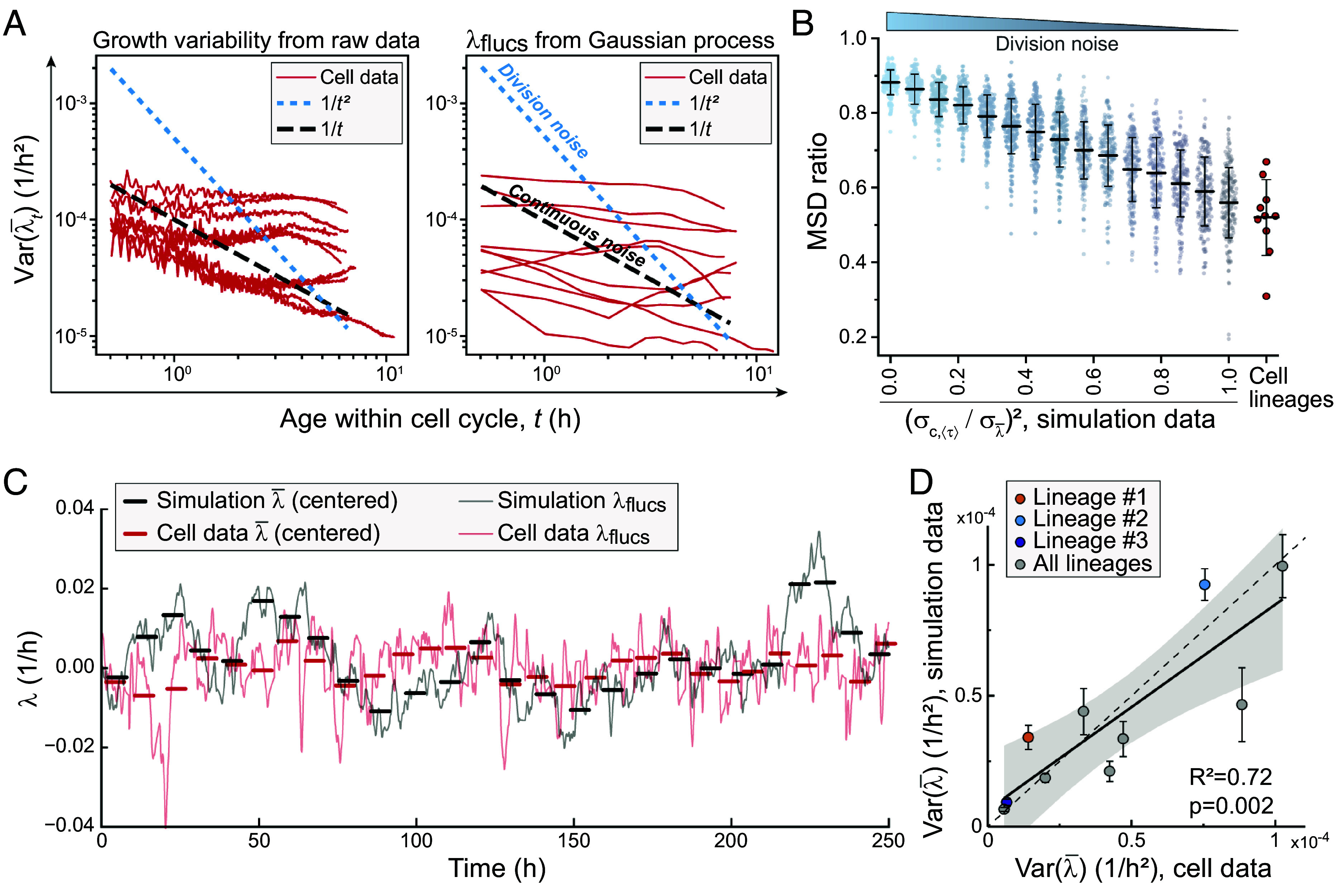
Cell-to-cell growth variability arises predominantly from continuous growth fluctuations (*A*) Scaling of $\mathrm{var}({\overset{¯}{\lambda }}_{t})$ with age (t) in raw data (*Left*) and GP-isolated $\lambda_{\mathrm{flucs}}$ (*Right*). Each red line depicts a lineage. Dotted lines depict model predictions. (*B*) MSD ratio in simulated datasets (teal/gray) and in cell data (red). Simulated data based on the levels of $\sigma_{c,\langle \tau \rangle }/\sigma_{\overset{¯}{\lambda }}$. High $\sigma_{c,\langle \tau \rangle }/\sigma_{\overset{¯}{\lambda }}$ indicates that continuous growth noise dominates over cell division noise. Line and error bars represent mean ± SD, dots depict separate lineages. (*C*) An example of the $\lambda_{\mathrm{flucs}}$ in lineage #1 (continuous red line) and in a corresponding OU model simulated data, where growth is “blind” to cell divisions (continuous black line). Thick horizontal bars indicate $\overset{¯}{\lambda }$ for each cell. Data are normalized to mean-zero. (*D*) Correlation of cell-to-cell growth variability between the cell data and the simulated data with continuous OU noise. Each dot depicts a separate lineage. Error bars depict simulation SEM. Black line and shaded area depict linear fit and 95% CIs. Pearson correlation and *P*-value are also displayed. The dotted line indicates x=y. In all panels, N=10 longest lineages for cell data.

We compared these expressions with the experimental data by examining the scaling between $\mathrm{var}({\overset{¯}{\lambda }}_{t})$ and cell age (t) (Fig. 5*A*). We found that the scaling of the variance was more consistent with the OU model prediction compared with the division noise model, although in all lineages it decayed more slower than in the OU model. Note that this conclusion is based on the end of the cell-cycle where Eqs. **12** and **13** are valid approximations.

We next sought to use the lineage structure of our data to provide additional evidence regarding the sensitivity of cell growth fluctuations to cell division events. To this end, we generated simulated cell-size trajectories with $\sigma_{\overset{¯}{\lambda }}$ fixed and graded levels $\sigma_{c,\langle \tau \rangle }/\sigma_{\overset{¯}{\lambda }}$, where ⟨τ⟩ is the average generation time. We then added experimental noise and ran these through the GP pipeline. To compare the simulated data from these models with the experimental L1210 cell data, we calculated the mean squared displacement (MSD) of $\lambda_{\mathrm{flucs}}$ within each cell, $MSD_{\mathrm{within}}$, by calculating the squared difference in $\lambda_{\mathrm{flucs}}$ values across time lags restricted to individual cell cycles. Separately, we computed $MSD_{\mathrm{between}}$ by calculating the squared difference in $\lambda_{\mathrm{flucs}}$ values across equivalent time lags between different cells. The MSD ratio, $MSD_{\mathrm{between}}$/($MSD_{\mathrm{within}}$+$MSD_{\mathrm{between}}$), separates the simulated datasets with different values of D, which tunes the OU noise (Fig. 5*B*). This allows us to separate the contributions of continuous and division noise. Using the MSD ratio, we observed that the cell data is indistinguishable from the limiting case simulations where $\sigma_{z}=0$ (Fig. 5*B*). Thus, growth noise is not introduced at cell divisions but rather continuously throughout the cell cycle, akin to an OU process.

Finally, to test whether a simple OU model predicts the cell-to-cell growth variability within cell lineages, we fitted the parameters in the OU model using a loss function that depends only on fluctuations within the cell cycle. In particular, we fit γ and D by matching the variance of $A_{t}=\int_{0}^{t}\lambda_{\mathrm{flucs}}\left(s\right)ds$ between the model and the data, since $A_{t}$ has smaller error bars than $\lambda_{\mathrm{flucs}}$. Using the fit parameters for each lineage, we simulated lineages with a similar number of cells and time resolution as our experimental data (Fig. 5*C*). We then compared the predicted cell-to-cell variability in $\left(\overset{¯}{\lambda }\right)$ in the simulated lineages with the experimental data, and we observed a strong correlation (Pearson $R^{2}=0.72$, Fig. 5*D*). We conclude that cell-to-cell variability in $\overset{¯}{\lambda }$ can be largely explained by the instantaneous growth rate fluctuations, which are independent of cell divisions and size.

## Discussion

Here, we have elucidated the structure of cell growth by monitoring single leukemia cell mass accumulation rate across ancestral lineages. The high precision and temporal resolution of our data have allowed us to isolate overlapping but independent growth behaviors. We have verified that the growth behaviors we observe cannot be explained by technical measurement fluctuations, and our experiments are carried out under steady external conditions and across multiple independent setups. Additionally, our buoyant mass measurements are insensitive to fluctuations in cellular water content and thus distinct from experiments tracking cell volume growth over time. Thus, our results reflect predominantly cell-intrinsic behaviors in biomass growth. However, our work is not without limitations, and we discuss these in *SI Appendix*, section 4.

Our study provides insights into cell-size homeostasis on the level of ancestral cell lineages. We observed that size homeostasis is maintained using an adder phenomenology at the individual lineage level and that the correlations between initial and final cell sizes are achieved by regulating cell cycle duration rather than cell growth. In contrast, previous studies have reported that growth regulation is involved in size homeostasis in many, although not all, examined cell lines (5, 43). Importantly, a previous study using SMR data (25) indicated that L1210 cells, which expressed a fluorescent cell cycle reporter, rely on growth regulation for their cell size homeostasis (5), unlike our wild type L1210 cells. The difference between the previous study and our results may originate from minor differences in experimental setups or biological differences, possibly caused by the expression of an exogenous protein or the associated clonal selection. Consistent with the latter option, the role of growth regulation in cell-size homeostasis has also been shown to change upon expression of a fluorescent cell cycle reporter in the HT29 adenocarcinoma cells (5). Regardless of the underlying cause of these differences, these results suggest homeostatic plasticity in the mechanisms of cell-size control.

A key achievement of our study is the characterization of growth rate fluctuations and their influence on cell-to-cell growth variability within ancestral lineages. We found that this cell-to-cell growth variability within ancestral lineages reflects approximately 1/3 of all cell-to-cell growth variability in our dataset, with the remaining variability reflecting lineage-to-lineage differences. Curiously, the growth fluctuations were independent of cell size in our model system. To avoid overfitting, we favored the simplest description of the growth fluctuations which could capture the correlation structure within and across cell divisions. Our simple two-parameter model was obtained by adding perturbations to the growth rates at divisions on top of an Ornstein-Uhlenbeck (OU) model. With this modified OU model, we found, using two distinct approaches, that the growth dynamics are consistent with the limiting case of our two-parameter model where growth fluctuations are “blind” to cell division. Notably, modeling studies on imperfect cell divisions have suggested that partitioning errors introduce significant cell-to-cell variability (4, 13, 32, 33), and it would be reasonable to assume that this extends to cell growth rates. Indeed, recent studies in bacteria (30, 57) and mammalian cells (36) have revealed that cell volume growth fluctuations are larger in newborn cells than later in the cell cycle, suggesting that cell divisions may introduce growth noise. Our results on cell mass growth argue against this conclusion. The separate regulation of cell volume and cell mass growth may explain these discrepancies (24), but we also note that our cell line displays less cell mass division asymmetry than many other mammalian cell lines (50).

More broadly, our work shows that long-term cell-to-cell growth variability within a cell lineage is predominantly a byproduct of continuous short-timescale stochasticity in growth when cells rely on cell-intrinsic growth regulation. In a simplistic and hypothetical model, where growth rates are set by a single growth limiting-molecule, our results suggest that this growth limiting-molecule is either very high in abundance or that cells possess mechanisms that limit partitioning errors of the molecule. Cells have mechanisms to promote the symmetric partitioning of certain cell cycle regulators (58, 59) and cell organelles (60), making it plausible that key growth regulators also undergo similar partitioning.

## Materials and Methods

### Cell Culture Conditions.

The L1210 cells were obtained from ATCC (#CCL-219) and validated negative for *mycoplasma*. All SMR experiments and the maintenance of cell cultures were carried out in RPMI 1640 medium (Invitrogen, #11875093), supplemented with 10% heat inactivated FBS (Sigma-Aldrich, #F4135, Lot#13C519), 10 mM HEPES (Invitrogen, #15630080), 1 mM sodium pyruvate (Invitrogen, #11360070) and 1x Antibiotic-Antimycotic (Invitrogen, #15240112). All experiments were started when cells were at nonconfluent and exponentially growing concentration (100.000 to 500.000 cells/ml).

Cell cycle and proliferation status were examined by fixing cells with 4% PFA for 10 min, permeabilizing cells with 0.25% Triton X-100 for 10 min, washing and blocking the cells with PBS supplemented with 5% BSA for 15 min. The cells were labeled for Ki-67 with 1:250 diluted Alexa Fluor 488 conjugated anti-Ki-67 rabbit monoclonal antibody (Cell Signaling Technologies, #11882) overnight, washed with PBS supplemented with 5% BSA for 15 min, then labeled for DNA with FxCycle PI/RNase staining solution (Invitrogen, #F10797) for 30 min. The cells were analyzed using a flow cytometer (LSR II HTS, BD Biosciences, 488 nm and 561 nm excitation lasers, 530/30 and 610/20 emission filters).

### SMR Operation.

The SMR chip was mounted on a metal holder connected to a 37 ^°^C water bath to maintain constant temperature. The cells were loaded into the SMR from vials pressurized with 5% CO2 and 21% O2. The fluidic pressure system was set to flush fresh media into SMR with every cell measurement, approximately every 1 min, thereby maintaining steady conditions throughout the experiment. Full details of the SMR setup, operation, and frequency data analysis can be found in refs. 27, 38, and 51.

The following changes were implemented to increase the long-term stability of the single-cell hydrodynamic trap: 1) Fresh media in SMR was replenished directly from 20 ml glass Wheaton vials to minimize the fluid-height driven pressure difference during the trap. 2) The glass Wheaton vials were placed on micrometers to tune fluid height daily throughout the experiments. 3) The glass Wheaton vials were not heated as heating accelerates water evaporation from the media. 4) Immediately after a cell was loaded into the SMR, we flushed other cells out of the SMR and the tubing using a flow rate of more than 10 nl/s and taping of the tubing for approximately 2 min. 5) After the initial cell loading step, the SMR was kept in the dark to remove any potential phototoxicity.

Across the study, three independent SMR setups were used to collect the data and no systematic differences were observed between the results of each setup.

### System Calibration.

The SMR systems were calibrated using two approaches (27, 38, 51). First, the SMR was loaded with sodium chloride solutions (0, 2, 6, 10, and 16% w/v) for which fluid densities are known. The SMR resonant frequency was measured at RT using open-loop setting to generate a baseline solution density calibration curve. Second, the change in SMR resonant frequency to an object of known buoyant mass was calibrated to derive a mass calibration factor (Hz/pg). 10 μm diameter polystyrene beads of density 1.05 g/cm^3^, suspended in DI water or PBS, were used as calibration particles.

### Autocorrelations.

Autocorrelation coefficients were obtained by using the following equation.

[14] $$\begin{array}{c} ACF\left(k\right)=\frac{1}{\left(N-k\right)\sigma^{2}}\sum_{n=1}^{N-k}\left(X_{n}-\mu \right)\left(X_{n+k}-\mu \right), \end{array}$$

where $X_{n}$ is the data (i.e. either λ or $\overset{¯}{\lambda }$). N and σ are the total number of data points and the SD of $X_{n}$, respectively. For plotting the ACF of λ as a function of time, we normalized the discrete time step between adjacent data points of the above equation by the median value of the time difference between the adjacent data points in our measurement or simulation ($t=kx_{\mathrm{cal}}$, where $x_{\mathrm{cal}}=median\left(\Delta t\right)$).

### Mean Squared Displacement (MSD) Analysis.

The mean squared displacement (MSD) of the fluctuations in λ for a time lag τ is defined as below

[15] $$\begin{array}{c} MSD\left(\tau \right)=\frac{1}{N}\sum_{t=t_{0}}^{t_{f}-\tau }{\left(\lambda \left(t+\tau \right)-\lambda \left(t\right)\right)}^{2}, \end{array}$$

where N is the total number of data summed between the first time point $t_{0}$ and the last time point $t_{f}$ subtracted by the time lag τ. MSD was computed for each lineage.

The $MSD_{\text{within}}$ of the fluctuations in $\lambda_{i}$ was calculated by only computing the MSD values within each cell i in the lineage. In other words

[16] $$\begin{array}{c} MSD_{\text{within}}\left(\tau \right)=\frac{1}{k}\sum_{i}\left(\frac{1}{N_{i}}\sum_{t=t_{0,i}}^{t_{f,i}-\tau }{\left(\lambda_{i}\left(t+\tau \right)-\lambda_{i}\left(t\right)\right)}^{2}\right), \end{array}$$

where k is the total number of cells within the lineage, $N_{i}$, $t_{0,i}$ and $t_{f,i}$ are the total number of data summed, the first time point and the last time point of the data, respectively, for individual cell i in the lineage.

The $MSD_{\text{between}}$ of the fluctuations in $\lambda_{i}$ and $\lambda_{j}$ was calculated by only computing the MSD values across different cells. In other words

[17] $$\begin{array}{c} MSD_{\text{between}}\left(\tau \right)=\frac{1}{N_{\text{between}}}\sum_{i}\left(\frac{1}{N_{i}}\sum_{t=t_{0,i}}^{t_{f,i}-\tau }{\left(\lambda_{j}\left(t+\tau \right)-\lambda_{i}\left(t\right)\right)}^{2}\right), \end{array}$$

where j≠i. Note that for each cell i, there is at most one other cell j for which the deviation $\lambda_{j}\left(t+\tau \right)-\lambda_{i}\left(t\right)$ can be calculated. This limitation arises due to the sequential nature of our dataset, where $t_{f,i}<t_{0,j}$ if i<j.

The MSD ratios represent

[18] $$\begin{array}{c} MSDratio=\frac{\left\langle MSD_{\text{between}}\right\rangle }{\left\langle MSD_{\text{between}}\right\rangle +\left\langle MSD_{\text{within}}\right\rangle }, \end{array}$$

where ⟨MS⟩ indicate values averaged for τ between 0 h and 4 h.

## Supplementary Material

Appendix 01 (PDF)

Dataset S01 (XLSX)

## Data Availability

Data are a combination of our previously published data (7 lineages) (27) and new experimental data (17 lineages), all collected under identical conditions. All single-cell mass traces can be found attached in the online supporting information (Dataset S1). Code for the simulations and data analysis can be found at https://github.com/elevien/L1210/tree/published. Analysis code data have been deposited in GitHub (https://github.com/elevien/L1210). Previously published data were used for this work (17, 27). All single-cell mass traces can be found attached in the online supporting information (*SI Appendix*, Table S1). All other data are included in the manuscript and/or supporting information.
